# Andrographolide Attenuates Established Pulmonary Hypertension via Rescue of Vascular Remodeling

**DOI:** 10.3390/biom11121801

**Published:** 2021-11-30

**Authors:** Xiaowei Nie, Chenyou Shen, Jianxin Tan, Xusheng Yang, Wei Wang, Youai Dai, Haijian Sun, Zhiyuan Wu, Jingyu Chen

**Affiliations:** 1 Shenzhen People’s Hospital, Shenzhen Institute of Respiratory Diseases, Shenzhen 518055, China; 2 Shenzhen Key Laboratory of Respiratory Diseases, Shenzhen People’s Hospital (The First Affiliated Hospital, Southern University of Science and Technology), Shenzhen 518055, China; 3 Lung Transplant Group, Wuxi People’s Hospital Affiliated to Nanjing Medical University, Wuxi 214023, China; shenchenyou@163.com (C.S.); jxt_410@163.com (J.T.); yxs15190303797@163.com (X.Y.); weiya0622@163.com (W.W.); yad_11038@126.com (Y.D.); 4 Department of Pharmacology, Yong Loo Lin School of Medicine, National University of Singapore, Singapore 119077, Singapore; sunhaijian927@163.com (H.S.); funmangrove@gmail.com (Z.W.)

**Keywords:** andrographolide, pulmonary hypertension, vascular remodeling, pulmonary artery smooth muscle cells

## Abstract

Pulmonary hypertension (PH) is characterized by vascular remodeling caused by marked proliferation of pulmonary artery smooth muscle cells (PASMCs). Andrographolide (ANDRO) is a potent anti-inflammatory agent which possesses antioxidant, and has anticarcinogenic activity. The present study examined potential therapeutic effects of ANDRO on PH in both chronic hypoxia and Sugen5416/hypoxia mouse PH models. Effects of ANDRO were also studied in cultured human PASMCs isolated from either healthy donors or PH patients. In vivo, ANDRO decreased distal pulmonary arteries (PAs) remodeling, mean PA pressure and right ventricular hypertrophy in chronic hypoxia- and Sugen/hypoxia-induced PH in mice. ANDRO reduced cell viability, proliferation and migration, but increased cell apoptosis in the PASMCs isolated from PH patients. ANDRO also reversed the dysfunctional bone morphogenetic protein receptor type-2 (BMPR2) signaling, suppressed [Ca2^+^]_i_ elevation, reactive oxygen species (ROS) generation, and the upregulated expression of IL-6 and IL-8, ET-1 and VEGF in PASMCs from PH patients. Moreover, ANDRO significantly attenuated the activation of TLR4/NF-κB, ERK- and JNK-MAPK signaling pathways and reversed the inhibition of p38-MAPK in PASMCs of PH patients. Further, ANDRO blocked hypoxia-triggered ROS generation by suppressing NADPH oxidase (NOX) activation and augmenting nuclear factor erythroid 2-related factor 2 (Nrf2) expression both in vitro and in vivo. Conventional pulmonary vasodilators have limited efficacy for the treatment of severe PH. We demonstrated that ANDRO may reverse pulmonary vascular remodeling through modulation of NOX/Nrf2-mediated oxidative stress and NF-κB-mediated inflammation. Our findings suggest that ANDRO may have therapeutic value in the treatment of PH.

## 1. Introduction

Pulmonary hypertension (PH) is a rare disorder characterized by remodeling of the small pulmonary arteries (PAs) and increased pulmonary arterial pressure, ultimately leading to progressive right heart failure and death [1]. The remodeling process involves interaction among all cell types (e.g., endothelial cells, smooth muscle cells, fibroblasts) present in the distinct layers of the pulmonary arteries causing histological changes to the pulmonary vessel wall. Endothelial dysregulation and proliferation in the intima, smooth muscle cell proliferation, and resistance to apoptosis within the medial layer, along with adventitial fibroblast activation, cause the remodeling of the pulmonary vessels. These events narrow the vascular lumen and therefore cause pulmonary hypertension [2]. Therefore, inhibition of cell proliferation or induction of cell apoptosis to reverse pulmonary remodeling may be an effective therapeutic strategy for PH. Emerging evidence indicates that PH may be considered as a proliferative disorder with a cancer-like nature and shares a large number of underlying pathogenic mechanisms with cancers [3]. This offers exciting opportunities to employ certain cancer-specific therapeutic strategies as well as anti-cancer agents for the treatment of PH [4].

In recent years, the use of traditional medicines for the prevention and treatment of human cancers has been increasing [5]. Andrographolide (ANDRO), a natural labdane diterpene lactone from *Andrographis paniculate*, was often used as traditional herbal medicine in many Asian countries for thousands of years. ANDRO possesses a wide range of pharmacological activities including anti-viral and anti-inflammatory effects [6,7]. In addition, ANDRO has shown great promise as an anti-cancer agent against a number of cancer cell types. ANDRO reduced the growth and cell apoptosis of breast tumor by inhibiting HIF-1 and its upstream PI3K/AKT signaling, and inducing G1 arrest [8]. The anti-proliferative activity of ANDRO in human cancers prompted us to investigate the potential therapeutic efficacy of ANDRO in the pulmonary vascular remodeling process of human PH.

ANDRO can also affect the NF-κB signaling, reactive oxygen species and nitric oxide synthase so that it implements a marked influence on the anti-inflammatory activity [9]. Nuclear factor-kappa B (NF-*κ*B) has been shown to play an important role in inflammatory reaction, immunological response, cell proliferation and apoptosis. Notably, NF-*κ*B expression is increased in rat PH models and contributes to the pathogenesis of hypoxic PH. Normally NF-*κ*B has no transcription activity due to the binding to the inhibitory factor (I*κ*B). Various stimuli promote the disassociation of NF-*κ*B from I*κ*B, leading to the translocation of NF-*κ*B active forms p50 and p65 to the nucleus where they regulate gene transcription [10,11,12,13]. The activation of the NF-*κ*B signaling pathway governs the transcription of pro-inflammatory cytokines, including tumor necrosis factor alpha (TNF-α), interleukin (IL)-6, and IL-1β [14,15,16]. All the above proinflammatory cytokines thus trigger vascular angiogenesis and remodeling. For this reason, targeting the NF-*κ*B pathway has been suggested as a useful strategy for controlling PH [17]. In addition, hypoxia augments the expression of ROS-generating NADPH oxidases, including NOX2, NOX4, and p47phox in PH [18]. Notably, hypoxia-induced oxidative stress is also closely tied to the NF-*κ*B-mediated inflammatory responses. Considering the complex crosstalk between these two events, a therapeutic agent with both anti-inflammatory and antioxidant activities is promising for the treatment of human PH.

## 2. Results

### 2.1. ANDRO Prevents the Development of PH in Hypoxia Mice

**Figure 1:**
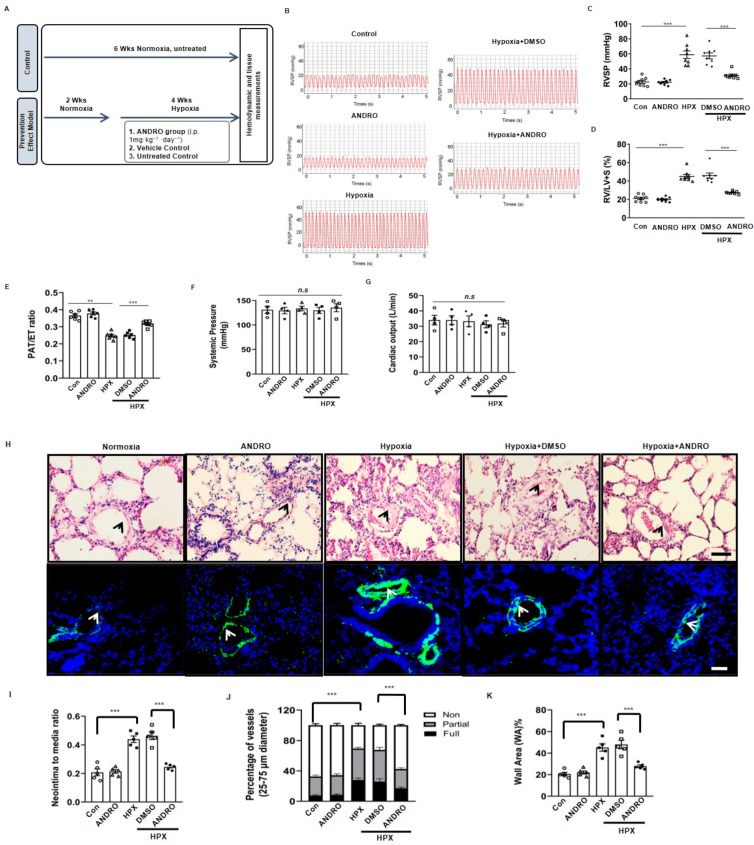
Andrographolide (ANDRO) prevented mice from hypoxia-induced pulmonary hypertension (PH). (**A**) Protocol of animal experiments. For hypoxia-induced PH model, mice were intraperitoneally injected with ANDRO (1 mg·kg^−1^·day^−1^) or a vehicle for two weeks followed by hypoxia for four weeks. Control mice were subjected to six weeks normoxia only. (**B**) Representative tracings showing the effect of ANDRO on pulmonary artery systolic pressure in hypoxia (HPX)-induced PH mouse model. *n* = 8. (**C**) ANDRO treatment significantly attenuated the elevated right ventricular systolic pressure (RVSP) in HPX-induced PH mice. Scale bars are 100 μm. *n* = 8. (**D**) ANDRO reversed right ventricular hypertrophy in HPX-induced PH in mice assessed invasively by the right ventricular hypertrophy index (RV/LV+S). *n* = 8. (**E**) Echocardiography analysis showing the decreased PAT/ET in hypoxia-induced PH mice were reversed by ANDRO. *n* = 6. (**F**,**G**) ANDRO failed to affect systemic pressure (**F**) and cardiac output (**G**). *n* = 8. (**H**) Representative H&E staining (upper panel) and α-SMA immunofluorescence staining (lower panel) images showing the vascular remodeling in various groups. (**I**) Quantitative analyses showing that ANDRO significantly reduced the ratio of neointima/media. *n* = 5. (**J**) Quantitative assessment of pulmonary arterial muscularization. Non-, partially and fully muscularized arteries as a percentage of total alveolar wall and duct arteries were scored (*n* = 5, one-way ANOVA for fully muscularized vessels). (**K**) ANDRO treatment significantly reduced the relative area of wall area (WA)%. *n* = 5. Data represent mean ± SEM. ** *p* < 0.01, *** *p* < 0.001. DMSO, Dimethyl sulfoxide; n.s.: no significant difference. Scale bars are 100 μm.

Mice were subjected to normoxia for two weeks, followed by hypoxia for four weeks (Figure 1A). As shown in Figure 1B–F, hypoxia for four weeks significantly increased right ventricular systolic pressure (RVSP)(Figure 1B,C) and the percentage of right ventricular hypertrophy index (RV/LV+S) ratio (Figure 1D), but had no significant effects on systemic pressure (Figure 1E) or cardiac output (Figure 1F). Treatment with ANDRO (1 mg·kg^−1^·day^−1^) for six weeks (two weeks before and four weeks during the hypoxia period) significantly attenuated the elevated RVSP or RV hypertrophy (Figure 1C,D).

H&E staining showed that ANDRO successfully reduced the percentage of medial thickness in small and medium-sized PA (Figure 1H upper panel and Figure 1I). Immunofluorescence staining of α-SMA also showed that ANDRO significantly attenuated the pulmonary arteriole muscularization in hypoxia-induced PH mice (Figure 1H lower panel and Figure 1J,K).

### 2.2. ANDRO Produces Therapeutic Effect on PH in Sugen-Hypoxia (SuHx) Mice

**Figure 2:**
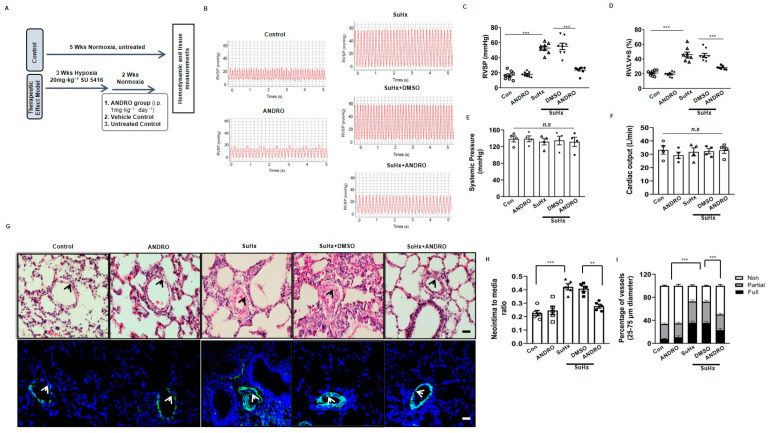

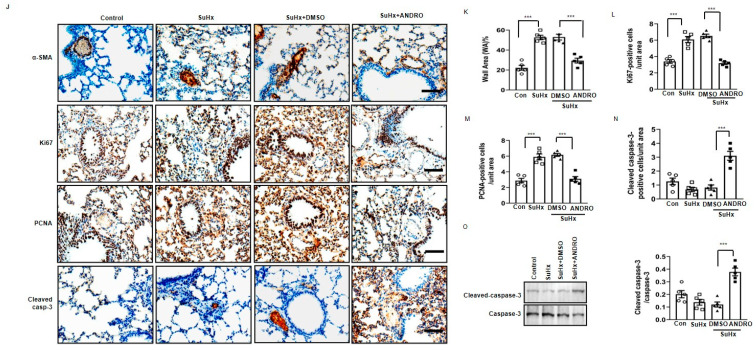
Therapeutic effects of ANDRO in a SU5416-hypoxia (SuHx)-induced mouse PH model. (**A**) Protocol of animal experiments. For SuHx-induced PH group, mice were subjected to three weeks hypoxia + Su5416 injection. ANDRO (1 mg/kg/day, i.p.) or DMSO (vehicle) were given for two weeks after three weeks of hypoxia + Su5416 injection. Control mice were subjected to five weeks normoxia only. *n* = 8. (**B**) Representative tracings showing the effect of ANDRO on PA systolic pressure in SuHx-induced PH mouse model. *n* = 8. (**C**) ANDRO treatment significantly attenuated the elevated RVSP in SuHx-induced PH mice. *n* = 8. (**D**) ANDRO reversed RV hypertrophy in SuHx-induced PH mice assessed invasively by the right ventricular hypertrophy index. (**E**,**F**) ANDRO failed to affect systemic pressure or cardiac output. *n* = 8. (**G**) Representative H&E staining (upper panel) and α-SMA immunofluorescence staining (lower panel) images showing the vascular remodeling in various groups. (**H**) Quantitative analyses showing that ANDRO significantly reduced the ratio of neointima/media. (**I**) Quantification of non-, partially and fully muscularized arteries as a percentage of total alveolar wall and duct arteries (*n* = 5, one-way ANOVA for fully muscularized vessels). (**J**–**O**) Representative immunohistochemical photomicrographs of serial lung sections and group data showing that ANDRO significantly reduced the increased expression of α-SMA (WA%, **K**), ratio of Ki67 positive cells (**L**) and ratio of PCNA positive cells (**M**) in SuHx-induced PH mice. ANDRO also significantly increased apoptosis, as reflected by cleaved-caspase-3 (**N**). This is further supported by Western blotting analysis (**O**). *n* = 5. Results are expressed in percent (%) calculated as follows: (number of positive cells divided by the total number of hematoxylin-stained nuclei) ×100, from six arteries per group. Scale bars, 100 μm. Data represent mean ± SEM. ** *p* < 0.01; *** *p* < 0.001; n.s.: no significant difference.

To determine the therapeutic potential of ANDRO, the SU5416-hypoxia (SuHx) mouse model of PH was used (Figure 2A). As shown in Figure 2B–D, ANDRO treatment (1 mg·kg^−1^·day^−1^) for two weeks significantly decreased RVSP (Figure 2C) and RV hypertrophy (Figure 2D), but had no significant effects on blood pressure (Figure 2E) and cardiac output (Figure 2F).

We also observed the effect of ANDRO on lung histology. H&E staining showed that ANDRO treatment reduced the thickened medial wall in small and medium-sized pulmonary vessels when compared with those treated with DMSO (Figure 2G upper panel and Figure 2H). Immunofluorescence (Figure 2G lower panel and Figure 2I) and immunohistochemical (Figure 2J upper panel and Figure 2K) staining of α-SMA further confirmed this result.

### 2.3. ANDRO Significantly Reduces Cell Proliferation and Increases Cell Apoptosis in PASMCs in SuHx-Induced PH Mice

Cell proliferation and apoptosis are two major processes of vascular remodeling in PH. It was found that ANDRO treatment reversed PH-induced increase of Ki67- (Figure 2J,L) and PCNA-positive cells (Figure 2J,M), suggesting that ANDRO can inhibit PASMCs proliferation in PH mice.

We also observed the effect of ANDRO on cell apoptosis with immunohistochemistry (Figure 2J lower panel and Figure 2N) and Western blots (Figure 2O). Both analyses showed that ANDRO increased the level of cleaved-caspase 3 (Figure 2O). These data suggest that ANDRO can stimulate apoptosis in PASMCs of PH mice.

### 2.4. Effect of ANDRO on PASMCs Isolated from PH Patients (PH-PASMCs)

**Figure 3:**
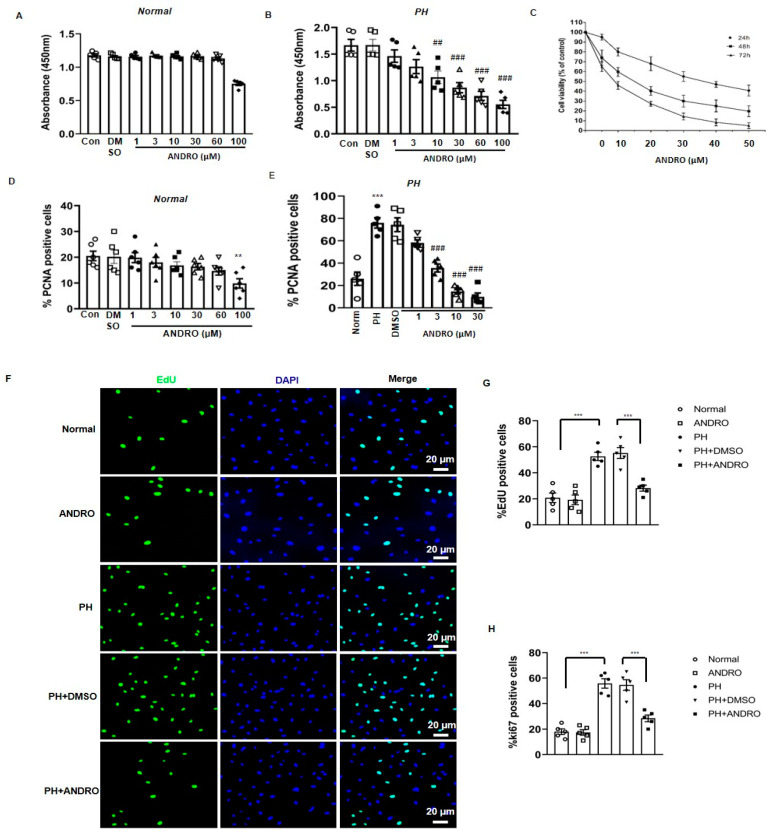
ANDRO reduced cell viability and proliferation of pulmonary artery smooth muscle cells from PH patients (PH-PASMCs). (**A**,**B**) Treatment with ANDRO for 24 h only significantly reduced cell viability of normal PASMCs at 100 μM**,** but caused a significant reduction in PAH-PASMCs at the concentration range of 1–100 μM. (**C**) The dose- and time-dependent effects of ANDRO on cell viability of PH-PASMCs. (**D**,**E**) Treatment with ANDRO for 24 h only significantly reduced cell proliferation of normal PASMCs at 100 μM**,** but produced obvious effect in PH-PASMCs starting from 1 to 100 μM. (**F**,**G**) Representative EdU staining (**F**) and group data (**G**) showing that ANDRO treatment (30 μM, 24 h) reversed PH-PASMCs proliferation. (**H**) Ki67 staining assay showing that ANDRO (30 μM, 24 h) reversed PH-PASMCs proliferation. Mean ± SEM, *n* = 5. ** *p* < 0.01, *** *p* < 0.001 vs. normal; ^##^ *p* < 0.01, ^###^ *p* < 0.001 vs. PAH-DMSO.

We also investigated the effect of ANDRO on the cell viability of PASMCs isolated from either healthy donors (Norm-PASMCs) or PH patients (PH-PASMCs). CCK-8 analysis showed that ANDRO at 1–60 μM had no significant effect on Norm-PASMCs (Figure 3A). However, at the same concentration range, it significantly reduced the cell viability in PH-PASMCs in a concentration-dependent manner (Figure 3B). The IC_50_ of the effect of ANDRO was at ~38 μM. For this reason, ANDRO at 30 μM was used in the subsequent cellular experiments if not specifically stated. Similarly, MTT assay also showed concentration-dependent decreases in cell viability when treated with ANDRO for 24, 48 or 72  h (Figure 3C).

ANDRO at 1–60 μM, had no significant effect on cell proliferation in Norm-PAMCs (Figure 3D), which itself had concentration-dependently reduced the percentage of PCNA positive cells in PH-PASMCs (Figure 3E). Similar results were also found in the EdU-(Figure 3F,G) or Ki67- (Figure 3H) positive cells when treated with ANDRO at 30 μM for 24 h.

**Figure 4:**
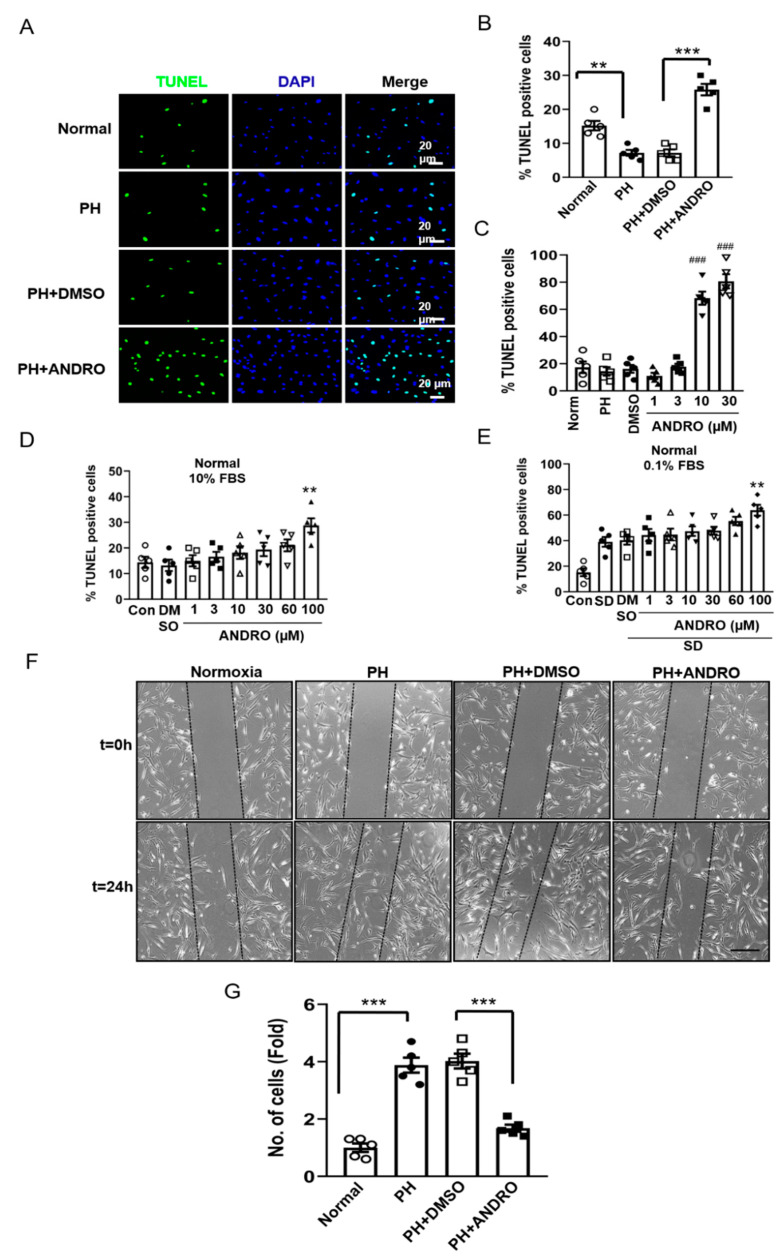
ANDRO reversed the resistance of cells to apoptosis and decreased migration of PH-PASMCs. (**A**,**B**) Representative TdT-mediated dUTP nick end labeling (TUNEL) assay image (**A**) and group data (**B**) showing that ANDRO reversed apoptosis resistance of PH-PASMCs when compared to the healthy control. (**C**) The dose-dependent effect of ANDRO (1–30 μM) on cell apoptosis in PH-PASMCs. (**D**,**E**) ANDRO only increased cell apoptosis at higher concentrations (60 μM) in healthy PASMCs, both under normal (10% FBS) (D) or serum deprivation (0.1% FBS) (E)conditions. Values are expressed as mean ± SEM, *n* = 5. ** *p* < 0.01, *** *p* < 0.001 vs. normal; ^###^ *p* < 0.001 vs. PH-DMSO. (**F**,**G**) Representative images of wound-healing assay (**F**) and group data (**G**) showing that ANDRO (30 μM, 24 h) significantly reversed cell migration in PH-PASMCs. *n* = 5. Data are shown as mean ± SEM. *** p < 0.001.

We also tested the effect of ANDRO on cell apoptosis in PH-PASMCs. As shown in Figure 4A,B, there was a significant decrease in the percentage of TdT-mediated dUTP nick end labeling (TUNEL)-positive cells in PH-PASMCs compared with that in Norm-PASMCs. Treatment with ANDRO significantly increased apoptotic cells in PH-PASMCs. The concentration-dependent effect of ANDRO was shown in Figure 4C. ANDRO at 1–30 μM concentration-dependently increased cell apoptosis in PH- PASMCs. However, for Norm-PASMCs, ANDRO only promoted cell apoptosis at higher concentrations in both 10% FBS (a condition that is known to promote proliferation) (Figure 4D) and 0.1% FBS (a “starvation” condition that promotes apoptosis) (Figure 4E) situations.

Cell migration was assessed with a wound-healing assay. There was a 4-fold increase in migration in PH-PASMCs when compared with that in normal control. This was significantly attenuated by treatment with ANDRO for 24 h (Figure 4F,G).

### 2.5. ANDRO Inhibits the TLR4/NF-κB Signaling Pathway in Human PH-PASMCs or PAs of PH Mice

Since the TLR4/NF-κB pathway is important in the development of PH, we investigated the involvement of this pathway in the therapeutic effect of ANDRO.

**Figure 5:**
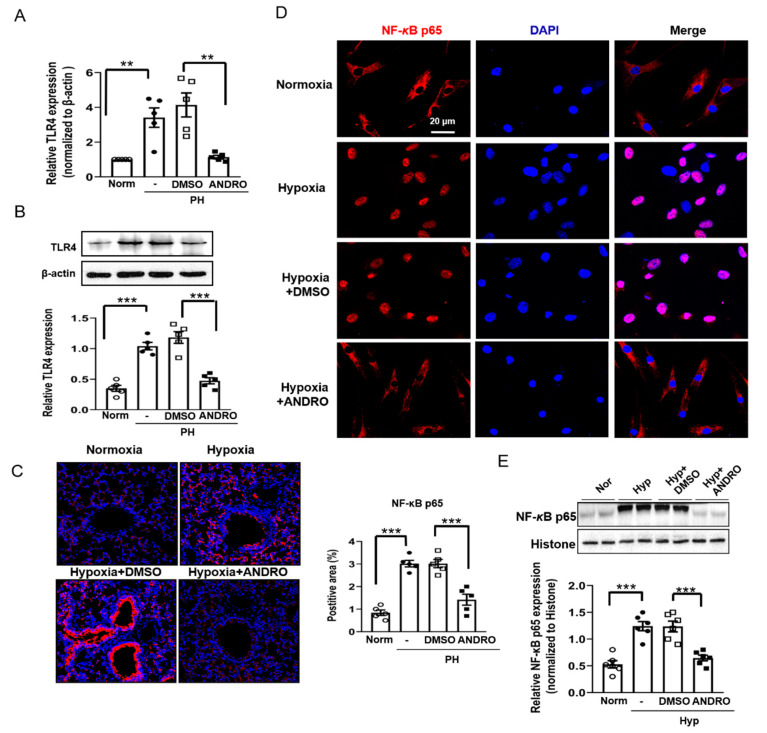
ANDRO suppressed the activation of NF-*κ*B in human PH-PASMCs or PAs of PH mice. (**A**,**B**) qPCR (**A**) and Western blotting (**B**) analysis showing that ANDRO (30 μM, 24 h) suppressed the mRNA and protein expression of TLR4. (**C**) Representative immunohistochemical staining and group data showing that ANDRO inhibited the expression of phosphorylated-NF-*κ*B p65 (Ser276) in the lung tissues of hypoxia-induced PH mice. Scale bars are 50 μm. (**D**,**E**) immunofluorescence (**D**) and Western blotting (**E**) analysis showing that ANDRO (30 μM, 24 h) reversed hypoxia-induced translocation of p65 to nuclei. Data represent mean ± SEM. *n* = 5, ** *p* < 0.01, *** *p* < 0.001.

As shown in Figure 5, the mRNA level (Figure 5A) and the protein expression (Figure 5B) of TLR4 were significantly upregulated in PH-PASMCs compared with those in the healthy controls. Treatment with ANDRO at 30 μM for 24 h, but not the vehicle (DMSO), significantly reversed the above effects.

To detect the involvement of the NF-kB pathway, we observed the phosphorylation of p65 (Ser276) protein in the lung section of PH mice and its translocation of p65 to nuclei in human PASMCs subjected to hypoxia. In the hypoxia mouse model, phosphor-p65 level was significantly upregulated in the PAs of the PH group (Figure 5C). In addition, both immunofluorescence (Figure 5D) and Western blotting (Figure 5E) analysis demonstrated that hypoxia induced activation of NF-kB in human PASMCs. Treatment with ANDRO reversed the above effects. Collectively, our data suggest that ANDRO may produce a therapeutic effect on PH via inhibition of the TLR4/NF-κB p65 pathway.

### 2.6. ANDRO Resumes NF-κB-Related Signaling Cascade in PH-PASMCs

**Figure 6:**
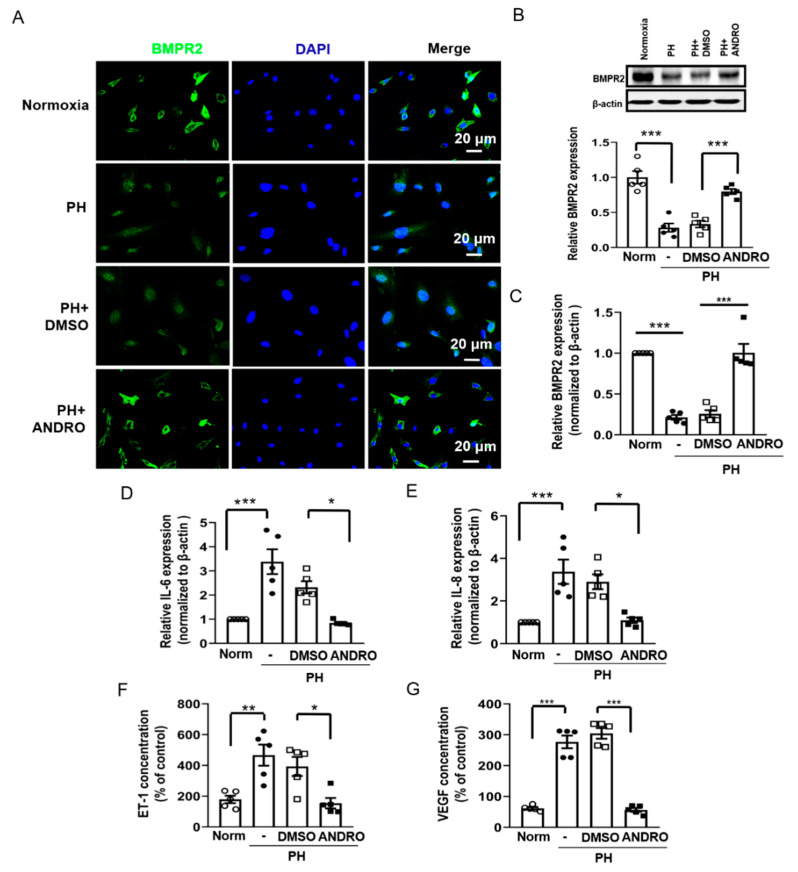
Effect of ANDRO on NF-*κ*B-related signaling cascade in PH-PASMCs. (**A**–**C**) Immunofluorescence staining (**A**), Western blots (**B**) and qRT-PCR (**C**) showing the effect of ANDRO on the expression of BMPR2 in PH-PASMCs. (**D**,**E**) QRT-PCR analysis showing that ANDRO significantly decreased IL-6 (**D**) and IL-8 (**E**) expression in PH-PASMCs. (**F**,**G**) ELISA assay showing that ANDRO significantly decreased ET-1 (**F**) and VEGF (**G**) expression in PH-PASMC. Data represent mean ± SEM. *n* = 5. * *p* < 0.05, ** *p* < 0.01, *** *p* < 0.001.

BMPR2, a protein regulated by NF-kB, plays an important role in the pathogenesis of PH. Next, we examined whether ANDRO enhanced BMP signaling in PASMCs. BMPR2 was localized predominantly to the cell membranes and cytoplasm of cultured human PASMCs. In PH-PASMCs, the expression of BMPR2 was significantly decreased, with some translocation of protein to the cell nucleus. We observed that ANDRO restored intracellular trafficking of BMPR2 (Figure 6A). Similar results were also found with protein and mRNA expression (Figure 6B,C) of BMPR2. These data suggest that ANDRO may produce therapeutic effects on PH via resuming the BMPR2 signaling.

We also studied the effect of ANDRO on the levels of IL-6 and IL-8, endothelin-1 and VEGF, all of which are important in pulmonary vascular remodeling. It was found that ANDRO reversed the up-regulated mRNA expression of both IL-6 (Figure 6D) and IL-8 (Figure 6E) in PH-PASMCs. Similarly, ANDRO also reversed the increased ET-1 (Figure 6F) and VEGF (Figure 6G) levels in PH-PASMCs. These data confirm that ANDRO can reduce pulmonary vascular remodeling in PH.

### 2.7. ANDRO Regulates Intracellular [Ca2^+^]_i_, ROS Production and the Related Signaling in PH-PASMCs

**Figure 7:**
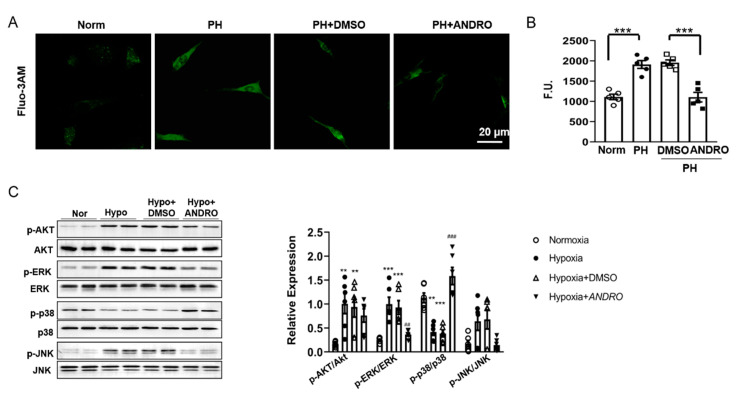
Effect of ANDRO on [Ca^2+^]_i_, and related signaling pathways. (**A**,**B**) Representative image (**A**) and group data (**B**) showing that ANDRO significantly reduced [Ca^2+^]_i_ elevation in PH-PASMCs compared to healthy PASMC (*n* = 18 from five independent experiments). Scale bars are 20μm. (**C**) Western blot analysis showing the effects of ANDRO on phosphorylation of AKT, ERK, p38 and JNK. *n* = 6. Data represent mean ± SEM. ** *p* < 0.01, *** *p* < 0.001 vs. control; ^##^ *p* < 0.01, ^###^ *p* < 0.001 vs. PH-DMSO.

Since [Ca^2+^]_i_ was found to be increased in PH-PASMCs, we detected whether ANDRO can reverse this effect using Fluo-3-AM as a [Ca2^+^]_i_ indicator. As expected, ANDRO decreased [Ca^2+^]_i_ in PH-PASMCs to a level similar to that in Norm-PASMC (Figure 7A,B).

As increased ROS production is one of the important characteristics of PH, we also determined ROS production using a fluorescent probe, 2′-7′ dichlorofluorescein diacetate (DCFDA) dye assay. As shown in Figure 7C, there was a significant increase in ROS generation in PH-PASMCs compared with that in Norm-PASMCs. Treatment with ANDRO at 1–30 µM for six hours concentration-dependently reduced ROS generation.

ROS-mediated activation of PI3K/Akt and MAPKs pathways are important in PH pathogenesis [19]. We also studied the effect of ANDRO on ROS-mediated signaling pathways. As shown in Figure 7D, hypoxia activated Akt, and ERK- and JNK-MAPKs, but inhibited p38-MAPK. Treatment with ANDRO reversed all these effects except Akt. These data suggest that ANDRO may produce beneficial effect via suppressing ERK-, JNK- MAPKs and activating p38-MAPK.

### 2.8. ANDRO Treatment Attenuates Hypoxia-Induced Pulmonary Oxidative and Nitrative Stress

**Figure 8:**
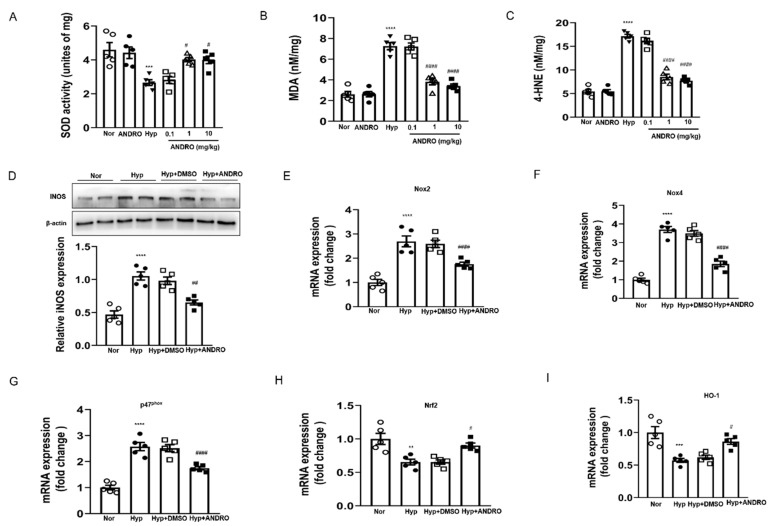
ANDRO treatment inhibited hypoxia-induced pulmonary oxidative/nitrative stress. (**A**–**C**) Effect of ANDRO on the levels of SOD (**A**), MDA (**B**), and 4-HNE (**C**) in homogenized fresh lung tissues. (**D**) Western blot analysis showing that ANDRO reversed the increased iNOS level in hypoxia-treated mice. (**E**–**G**) Real-time qPCR analysis showing that ANDRO treatment reduced hypoxia-upregulated mRNA expression of the ROS-generating NADPH oxidases (NOX2, NOX4, and p47 phox) in the lung tissues. (**H**, **I**) Real-time qPCR analysis showing that the down-regulated mRNA expressions of Nrf2 and HO-1 in the lung tissues were attenuated by ANDRO treatment. ** *p* < 0.01, *** *p* < 0.001, **** *p* < 0.0001, compared to normoxia control; ^#^ *p* < 0.05, ^##^ *p* < 0.01, ^####^ *p* < 0.001, compared to Hyp-DMSO.

Oxidative stress drives the development of PH. Reduced SOD activity (Figure 8A) along with augmented lipid peroxides (MDA, Figure 8B,C) were found in the hypoxia-induced PH lungs. However, these detrimental changes were attenuated by treatment with ANDRO (10 mg/kg/day, two weeks) during the course of PH. Western blot analysis showed that ANDRO treatment could inhibit the expression of inducible nitric oxide synthase (iNOS) in the PH lungs (Figure 8D). Real-time qPCR analysis indicated that ANDRO remarkably mitigated the expression of various ROS-generating NADPH oxidases (NOX2, NOX4, and p47phox) induced by PH (Figure 8E–G). Treatment with ANDRO also reversed the PH-induced downregulation of Nrf2 and HO-1 levels (Figure 8H,I). These data indicate that ANDRO treatment may abrogate hypoxia-induced pulmonary oxidative and nitrative stress.

### 2.9. ANDRO Treatment Blocks ROS Generation via Regulating NOXs/Nrf2 Redox Imbalance in Human PH-PASMCs

**Figure 9:**
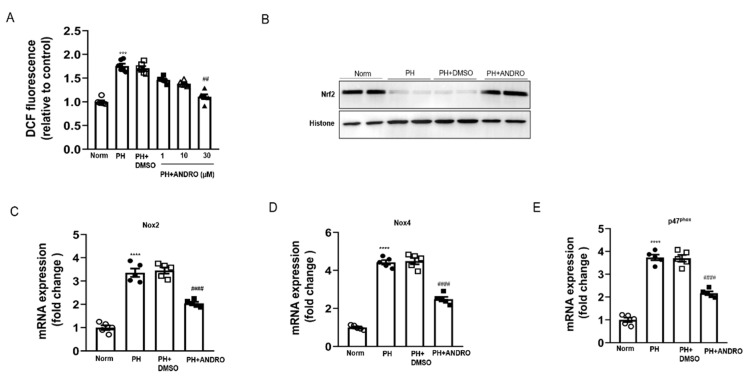
ANDRO treatment blocked ROS generation via regulating NOX/Nrf2 redox imbalance in human PH-PASMCs. (**A**) Intracellular ROS was measured with DCFH-DA probe using confocal microscopy. ANDRO at 30 μM attenuated ROS production in human PH-PASMCs. (**B**) Representative Western blot analysis showing that ANDRO treatment attenuated the reduced Nrf2 expression during PH. (**C**–**E**) Real-time PCR analysis showing that ANDRO treatment significantly attenuated the upregulated mRNA levels of NADPH oxidases (NOX2, NOX4, and p47 phox) in PH-PASMCs. *** *p* < 0.001, **** *p* < 0.0001, compared to healthy control; ^##^ *p* < 0.01, ^####^ *p* < 0.0001, compared to PH-DMSO.

We then explored the effect of ANDRO on PH-triggered ROS generation in vitro. Human PH-PASMCs induced a relatively rapid ROS generation compared with healthy controls, evidenced by the elevated fluorescence intensity of the 2,7-Dichlorodi -hydrofluorescein diacetate (DCFH-DA) and Dihydroethidium (DHE) (Figure 9A). As both NOX-mediated ROS production and the Nrf2-dependent antioxidant response participated in oxidative damage, we wonder whether NOXs/Nrf2 imbalance existed in PH-PASMCs. Simultaneously, we found that the expression of the Nrf2 was down-regulated (Figure 9B), while the expression of ROS-generating NADPH oxidases, including NOX2, NOX4, and p47phox, was significantly increased in PH-PASMCs (Figure 9C–E). ANDRO treatment decreased the expression of various NADPH oxidases (NOX2, NOX4, and p47phox). These results suggest that ANDRO attenuated PH-triggered ROS generation by inactivating NADPH oxidases and restoring Nrf2 expression in PH-PASMCs.

## 3. Discussion

PH is a progressive, debilitating and life-threatening disease [20]. The cellular and molecular pathological changes contribute to pulmonary vascular remodeling in PH, ultimately leading to increased pulmonary vascular resistance, right heart failure, and death [21]. Current therapies such as prostanoids, endothelin receptor antagonists, or phosphodiesterase5 inhibitors largely alleviate the symptoms of PH by promoting vasodilation [22]. However, these therapies do not attenuate PH progression. Moreover, they only have a limited impact on mortality [23]. Therefore, novel drugs are required urgently to produce better therapeutic effects on PH.

ANDRO has been reported to be of therapeutic effect for the treatment of various diseases for decades. In China, ANDRO are prepared as tablets in clinics for anti-bacterial, anti-inflammatory, upper respiratory tract infection and bacterial dysentery [24]. We demonstrated in the present study that ANDRO produces both preventive and therapeutic effects against PH in two independent PH models, namely the chronic and the SuHx models. ANDRO significantly improves PA pressure, PA medial hypertrophy, and RV hypertrophy, without affecting systemic pressure and cardiac outputs. Histology and immunostaining results also confirmed that ANDRO significantly attenuated the distal neomuscularization and reduced the medial thickness in small and medium-sized PA. Moreover, ANDRO treatment reduced cell proliferation and stimulated apoptosis of PASMCs in the lung tissues of PH mice. To reveal whether ANDRO can produce similar results in PH patients, we isolated PASMCs from both healthy donors and PH patients. Consistent with the findings in animal models, ANDRO also inhibited cell viability, proliferation and migration, but increased cell apoptosis in the PASMCs isolated from PA patients. However, ANDRO at the same concentration range only produced minor effects on healthy PASMCs. Our results highlight the possible efficiency of PH to treat the lethal pathology of PH.

In PH, hypoxia-induced ROS generation interacts with the NF-κB proinflammatory pathway. This, in turn, increases the levels of proinflammatory cytokines, cell adhesion molecules, and iNOS [25]. Therefore, oxidative stress, inflammatory pathways, and stress signaling are closely interrelated and eventually promote PH development [26]. We next studied the mechanisms involved in the therapeutic effects of ANDRO.

NF-κB is of great importance in PH development. Activation of NF-κB induces the expression of various genes encoding proinflammatory cytokines and chemokines, such as IL-1β and IL-6; therefore, NF-κB can promote vascular inflammation [27]. In human PH, NF-κB is activated in the PASMCs and macrophages derived from patients with idiopathic PH, and NF-κB inhibition ameliorates hypoxia-induced PH [28]. In addition, increased circulating growth factors and cytokines have been reported in PH, which also activate NF-κB [29]. We found in the present study that ANDRO reversed the translocation of p65 from cytosol to nuclei and inhibited the expression of IL-6 and IL-8 in hypoxia/PH. These data suggest that ANDRO produces significant anti-inflammatory effect in PASMCs in the condition of PH.

Activation of NF-κB can also be achieved by other mechanisms. In vivo, endothelial dysfunction and inflammation are recognized as some of the earliest abnormalities in PH, resulting in a well-recognized imbalance of endothelium-derived vasoactive factors—with increased vasoconstrictors, all of which lead to NF-κB activation [30]. The hypoxia-induced ROS generation also activates NF-κB signaling and other stress signaling pathways [31]. Activation of PI3K/Akt, ERK1/2- and JNK-MAPKs has been reported to be associated with activation of the NF-κB pathway [32]. In agreement with previous findings [33,34], we also demonstrated that ANDRO significantly suppressed ROS generation and the activation of the above signaling pathways except Akt, and therefore also partially contributed to the anti-inflammatory effects.

Due to the presence of binding sequence within TLR4 promoter region, the increase in TLR4 expression observed in PH-PASMCs has been attributed to NF-κB activation [35]. Once activated, NF-κB regulates multiple genes that might positively reinforce its own expression and activation. Therefore, both TLR4 and NF-κB might be critical integrators of multiple signaling pathways and their downstream effects might explain several important features of PH [36,37]. Consistent with the above results, we also found that both TLR4 and NF-κB related signaling (e.g., IL-6 and IL-8) were upregulated in our models and treatment with ANDRO reversed these effects. These data confirm that ANDRO produced significant anti-inflammatory effects in PH-PASMCs via different mechanisms.

Activation of NF-κB induces the expression of various genes, including ET-1 and VEGF expression. ET-1 is a potent vasoconstrictor peptide, which was originally isolated from endothelial cells [38]. VEGF is an important factor that contributes to vascular remodeling in the development of PH. ET-1 and VEGF is upregulated in the lungs of PH patients [39]. Downregulation of BMPR2 signaling has been implicated in the development of experimental PH [40,41]. Activation of NF-κB has been recognized to reduce BMPR2 expression [42]. Our results also showed the expression levels of VEGF, ET-1 and BMPR2 reflected the severity of the PH, and ANDRO treatment attenuated the upregulated expression of VEGF and ET-1, and rescued the oxidative damage under hypoxic conditions [43].

Oxidative damage under hypoxic conditions is considered to be an important event in PH [43]. We found in the present study that both hypoxia and PH induced ROS generation via activation of NADPH oxidases (NOX2, NOX4, and p47phox). By inactivating NADPH oxidases, ANDRO attenuated PH-triggered ROS generation. NF-κB and ROS generation may interact with each other. Hypoxia-induced ROS generation activates various stress signaling pathways, including NF-κB signaling, which, in turn, also induces oxidative damage in PH [44]. In addition to inhibition of the above ERK1/2 and JNK pathways, we also found that ANDRO stimulated the suppressed p38-MAPK. This is consistent with previous findings and may be mediated by induction of the Nrf-2-HO-1 pathway [45,46]. Our data suggests the importance of the NF-κB axis in PH. For this reason, NF-κB can be considered as an integrator of multiple pathways implicated in PH, including ROS activation, BMPR2 downregulation and IL-6 expression [47]. Thus, we believe that the ANDRO-dependent inhibition of NF-κB could also explain its antioxidant effects.

**Figure 10:**
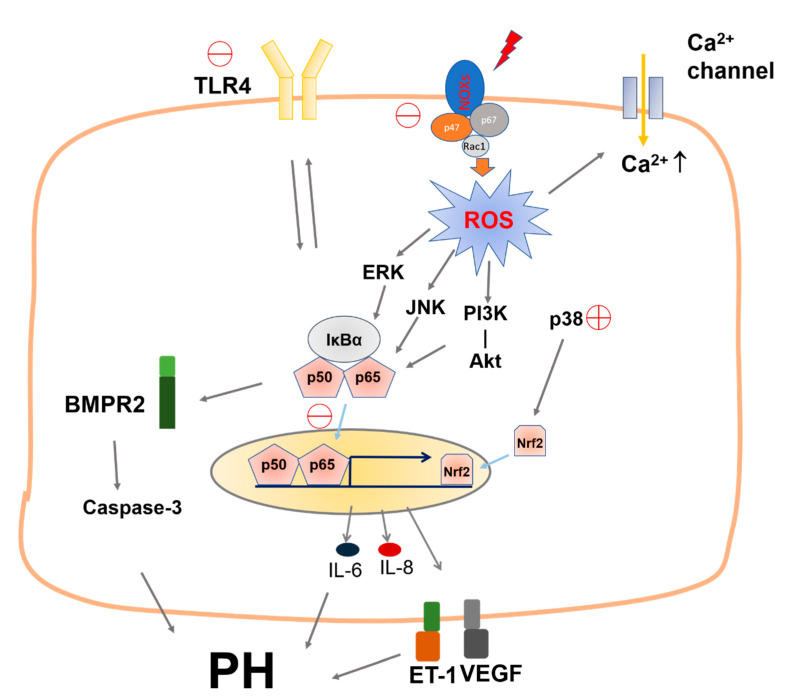
Schematic illustration of the mechanisms for the beneficial effects of ANDRO on PH. ANDRO inhibits ROS generation by decreasing NOX2, NOX4 and p47phox expression and suppresses the TLR4/NF-*κ*B pathway by inhibiting the protein expression of TLR4 and the translocation of NF-kb. ANDRO upregulates BMPR2 expression and inhibits cytokines like IL-6 and IL-8 expression, and ET-1 and VEGF protein expression. ANDRO also inhibits ROS generation and [Ca^2+^]_i_ elevation and the activation of JNK-/ERK-MAPKs. Interestingly, ANDRO stimulates p38-MAPK which is suppressed during PH. All these events contribute to the therapeutic effects of ANDRO.

In summary, we proved in the present study the validity of our experimental design and reliability of the protective effects of ANDRO treatment on PH (Figure 10). We found that ANDRO can restore most of the molecular and cellular abnormalities observed in human PH-PASMCs, including [Ca2^+^]_i_ elevation, ROS generation, and the upregulated production/expression of IL-6 and IL-8, ET-1 and VEGF. All these changes contribute to enhanced cell proliferation and reduced cell apoptosis in human PH-PASMCs. The multiple targets effect of ANDRO explains how a single drug such as ANDRO can affect so many pathophysiological processes and be efficient in several experimental models. In addition, ANDRO, which has the advantages of multi-targeted efficacy, low toxicity and low cost, shows promise in recent research. Expensive medical costs are also the main causes of patients’ unmotivated mental behavior in clinical treatment. Limited therapeutic scheduling for PH can be attributed to insufficient drugs to some extent. ANDRO might be a potential drug for PH treatment and additional investigations are required to ensure that the concrete molecular targets mediate the protective effects of ANDRO on PH.

Though this study is the first to demonstrate ANDRO as a potential drug strategy for reversing established PH in two PH animal models, several questions remain unanswered. The human clinical samples used in this study are from PH patients secondary to pulmonary fibrosis. It has been reported that ANDRO can attenuate bleomycin-induced pulmonary fibrosis via inhibition of inflammation and oxidative stress [48]. Therefore, ANDRO has potent anti-fibrotic effects in lung fibroblasts via inhibition of the proliferation and myofibroblast differentiation of fibroblasts and subsequent ECM deposition. For the above reasons, we cannot exclude the possibility that the beneficial effects of ANDRO against the pathology of PH-PASMCs is partially mediated by its anti-fibrotic effects. Nevertheless, our findings provide strong evidence that ANDRO is suitable as a new therapeutic tool for PH. We believe that it will also lead to a better understanding of the regulation of cell proliferation and apoptosis by ANDRO, which will benefit many other human diseases such as cancers.

## 4. Innovation

Given that conventional pulmonary vasodilators have limited efficacy for the treatment of severe PH, novel drugs are urgently required to produce better therapeutic effects on PH. We found in the present study that ANDRO may have therapeutic value in the treatment of PH by reversing pulmonary vascular remodeling.

## 5. Materials and Methods

### 5.1. Materials

ANDRO and SU5416 were purchased from Sigma-Aldrich (St. Louis, MO, USA). It was dissolved in dimethylsulfoxide (DMSO) at 1% for in vivo and 0.1% for in vitro studies. DMEM culture medium and HBSS were from Invitrogen (Carlsbad, CA, USA).

### 5.2. Study Design

Power calculations were determined based on similar publications in the field. Animals were randomly assigned to the experimental groups. Following the principle of equal opportunity, the random number table method was used to assign each animal. Male and female animals were used in equal numbers for all experiments. No samples or animals were excluded from analysis. All quantifications were performed in a blind fashion.

### 5.3. Clinical Samples Collection

This study was approved by the Ethics Committee for the Use of Human Samples at the Nanjing Medical University (China), which was in accordance with The Code of Ethics of the Helsinki Declaration of the World Medical Association for experiments involving humans, and each subject gave written informed consent. Human lung tissue was obtained with written informed consent from ten patients undergoing lung transplantation at the Lung Transplant Group, Affiliated Wuxi People’s Hospital of Nanjing Medical University (Wuxi, China) (Supplemental Table S1) [49]. Healthy control samples were collected from 10 healthy donors not suitable for transplantation. Human PASMCs were isolated from the lung tissues of normal donors and patients with PH.

### 5.4. Animals and Experimental Protocols

All animal studies were reviewed and approved by the Ethics Committee of the Southern University of Science and Technology. This study was in accordance with the Guide for the Care and Use of Laboratory Animals published by the US National Institutes of Health (NIH Publication No.85-23, revised 1996). C57BL/6J mice weighing 18–22 g were obtained from Shanghai Laboratory Animal Center. In general, mice were maintained in ventilated cages (five mice per cage) under specific pathogen-free conditions in a room at 20–24 °C, and relative humidity (40–70%) on a 12 h light/dark cycle with water and food ad libitum. Mice were allocated upon arrival into groups using restricted randomization so that average body weights were similar between the groups. In total, 64 mice (*n* = 8 each group) were included in this study, and all efforts were made to minimize suffering. Body weights in fed state were recorded during the course of the study to follow the wellbeing of the animals. Experiments were terminated if body weight decreased by 15% and/or if animals showed signs of distress, such as decreased movement, piloerection, dull eyes or abnormal posture.

To establish animal models of PH, mice were subjected to either chronic hypoxia alone or a combined chronic hypoxia with i.p. injection of semaxanib (SU 5416). After the experiments, mice were anaesthetized for hemodynamic and echocardiography measurements as previously described (Bellofiore et al., 2017). Lung tissues were prepared for haematoxylin and eosin (H&E) staining and immunohistochemistry, and heart tissues were fixed for vascular hyperplasia and right ventricular hypertrophy.

#### 5.4.1. Chronic Hypoxia Model of PH

Mice were randomly assigned into four groups (*n* = 8 each group). PH model mice were established as previously described [50] with modification and illustrated in Figure 1A. Briefly, (i) control group: mice were maintained in a chamber with normal air for 6 weeks; (ii) PH group: mice were maintained for 2 weeks in normoxia followed by hypoxic challenge for 4 weeks; (iii) vehicle control group: mice were intraperitoneally injected (i.p) with 1% DMSO once a day for 2 weeks before and during 4 weeks of hypoxic challenge; (iv) ANDRO group: mice were i.p with ANDRO (1 mg·kg^−1^·day^−1^) for 2 weeks before and during 4 weeks of hypoxic challenge. Hypoxia was generated using a chamber in which the oxygen concentration was maintained at 10% by controlling the flow rates of compressed oxygen and nitrogen. Normoxic animals were kept at 21% O_2_, adjacent to the hypoxic chamber in the same room.

#### 5.4.2. SuHx-Induced PH Murine Model

For the SuHx model of PH, mice (*n* = 8 each group) were injected with the vascular endothelial growth factor Flk-1/KDR receptor inhibitor SU 5416 (20 mg·kg^−1^) once weekly and kept in hypoxia (10% O_2_) for 3 weeks [36], as illustrated in Figure 2A. After the three weeks of hypoxia and weekly injections of SU 5416, animals were maintained in normoxia for an additional 2 weeks. In those two weeks, treatment groups received either DMSO as a vehicle control or ANDRO (1 mg·kg^−1^) daily by i.p. injection.

### 5.5. Hemodynamic Studies and Evaluation of Right Ventricular Hypertrophy

A 1.4F Millar catheter (Millar Instruments, Houston, TX, USA) was inserted into the right ventricle (RV) via the right jugular vein to measure the right ventricular systolic pressure (RVSP). The catheter was connected to a PowerLab Data Acquisition system and Lab Chart 7 software (AD Instruments, Spechbach, Germany) to record the data [51]. RV hypertrophy was assessed by the ratio of the weight of the right ventricle to that of the left ventricle (LV) and septum (S) [52].

### 5.6. Immunohistochemical Staining

To determine whether ANDRO reduces PA remodeling in hypoxia-PH mice, we measured PA media wall thickness. Lung tissue specimens were fixed in 4% paraformaldehyde (pH 7.4) for 24 h at room temperature. Lungs were prepared as paraffin-embedded tissue samples and cut into 4-µm-thick sections. After deparaffinizing, some sections were mounted on glass slides, rehydrated with distilled water and stained with H&E. Antigen retrieval was achieved by treating the sections with Immunosaver (Nisshin EM Co., Ltd., Tokyo, Japan) at 98 °C for 45 min in an electric cooking pot (WMF, Hayingen, Germany). After blocking the endogenous peroxidase activity with methanol containing 0.3% hydrogen peroxide for 30 min, the sections were treated with normal goat serum for 30 min, and then incubated with primary antibodies at 4 °C overnight.

Immunohistochemical staining was conducted using antibodies against α-smooth muscle actin (α-SMA) (1:200; Sigma-Aldrich Cat# A5228, St. Louis, MO, USA), proliferating cell nuclear antigen (PCNA; 1:500; Abcam Cat# ab2426, Cambridge, UK), Ki67 (1:200, Cell Signaling Technology Cat# 12202), and cleaved caspase-3 (1:1000; Abcam Cat# ab2302, Cambridge, UK) as primary antibodies. The HRP-DAB Cell & Tissue Staining Kits were used to detect staining of the primary antibody in accordance with the manufacturer’s instructions. Mayer’s hematoxylin (Sigma-Aldrich, Merck KGaA, Darmstadt, Germany) was used for counterstaining. Negative control sections for the immunohistochemical staining received identical treatments except for the absence of primary antibody, and they did not show any specific staining. Sections were visualized through an Olympus BX-51 microscope (Olympus, Tokyo, Japan) with a Retiga 4000RV camera (Q Imaging, Vancouver, Canada). Positive cells were quantified and normalized to total tissue area using ImageJ [53].

Assessment of pulmonary arteriolar muscularization and the subsequent categorization of the accompanying intra-acinar artery as non-, partially or fully muscularized, was judged by the degree of immunostaining for α-SMA. A minimum of 25 vessels with diameters ranging from 25 to 75 µm were counted from nonserial lung sections and categorized as either fully, partially or non-muscularized. Statistical significance was assessed by comparing the percentage of fully muscularized vessels between groups. Assessment of pulmonary arterial muscularization was performed in a blinded fashion [54].

### 5.7. Cell Culture

Primary human PASMCs were harvested from explanted lungs of patients undergoing transplantation for PH, or from unused lung tissue from donor control subjects. Briefly, human PAs were digested in Hanks’ balanced salt solution (HBSS) containing 2 mg·mL^−1^ type II collagenase (Worthington Biochem, Lakewood, NJ, USA) and 20 U·mL^−1^ DNase. Upon the removal of the endothelial layer, the remaining smooth muscles were digested in HBSS containing 2 mg·mL^−1^ collagenase, 0.5 mg·mL^−1^ elastase, and 2 mg·mL^−1^ BSA at 37 °C to make a cell suspension of PASMCs. Isolated PASMCs were resuspended in DMEM containing 10% fetal bovine serum (FBS), 100 IU·mL^−1^ penicillin, 100 μg·mL^−1^ streptomycin and incubated at 37 °C in a humidified atmosphere (5% CO_2_ in air). The purity of PASMCs was confirmed by the specific monoclonal antibody against α-SMA. Cell viability (usually >98%) was determined by Trypan Blue exclusion. Cells were used at passages 3–6. In vitro hypoxia experiments were performed in primary human PASMC (PromoCell, C12521, Heidelberg, Germany) obtained commercially. To induce hypoxia, cells were placed into a hypoxia chamber with an atmosphere of 5% CO_2_/92% N_2_, with an oxygen level at 3% for 48 h [55].

### 5.8. Cytotoxicity Assay

Exponentially growing human PASMCs were plated in flat-bottomed 96-well plates (Corning, New York, NY, USA) at 5 × 10^4^ cells/well. Serial dilutions of ANDRO were added to give a final volume of 200 μL·well^−1^. Control wells contained medium without ANDRO or vehicle (0.1% DMSO). Plates were incubated for different time points in a humidified 5% CO_2_ incubator and assayed for cell viability. MTT method was used to detect the 50% inhibitory concentration (IC_50_), which was defined as the concentration producing 50% decrease in cell growth. All determinations were confirmed in at least three independent experiments.

### 5.9. Cell Proliferation

EdU staining was performed using Click-iT 5-ethynyl-20-deoxyuridine (EdU) Imaging Kit according to the manufacturer’s protocol (Invitrogen Corporation, Carlsbad, CA, USA). After treatment with ANDRO or 0.1% DMSO as the vehicle control, human PASMCs were incubated with 10 μM EdU at 37 °C for 2 h. After three rinses with PBS, cells were permeabilized with 0.5% Triton X-100 for 10 min after fixing with 4% formaldehyde for 15 min at room temperature. Cells were then incubated with freshly made 1XClick-iT reaction cocktails overnight at 4 °C. PASMCs nuclei stained with DAPI were used for cell count and visualized using a laser scanning confocal microscope (Leica, Heidelberg, Germany). For quantification of PASMC proliferative rate, six randomly selected views from each sample image were used to calculate the relative EdU-positive ratio [56].

### 5.10. Detection of Reactive Oxygen Species (ROS)

Human PASMCs were seeded at 5 × 10^3^ cells per well in 96-well plates supplemented with 10% FBS DMEM for 24 h and allowed to attach. The media was then discarded, and the cells were washed by serum-free media, followed by the addition of 5% FBS DMEM media for 72 h with appropriate glucose and antioxidant concentrations, based on our intervention and the condition of each group. During the final 30 min of the assay, 2.0 µL of dihydroethidium (DHE) or 10 µL of 2′, 7′-dichlorfluorescein (DCF) were added to each well, and the cells were placed for 30 min in the dark before fluorescence microscopy was performed. DHE and DCF are fluorogenic dyes that measure ROS superoxide and hydrogen peroxide, respectively [57].

### 5.11. Enzyme-Linked Immunosorbent Assay (ELISA)

After the cells in each group were cultured for 24 h, an appropriate amount of cell lysate was taken, and the total protein level in the cell lysate was quantified using the Pierce BCA Protein Assay Kit (Thermo Fisher scientific, Pittsburgh, PA, USA). Enzyme-linked immunosorbent assay was performed to detect endothelin-1 (ET-1) and vascular endothelial growth factor (VEGF) in the culture supernatant of PASMCs of each group according to the manufacturer’s guidelines (R&D Systems Inc, Minneapolis, MN, USA) [58].

### 5.12. Cell Migration Assay

The migration of human PASMCs in vitro was assessed by means of a previously described wound healing assay [59]. Cells were plated at a density of 1 × 10^6^ cells on 4 cm^2^ culture dishes and incubated for 24 h. At this point, the cell monolayer was scratched with a pipet tip (P300). The medium and dislodged cells were removed and the plates were rinsed with PBS. Next, culture medium (DMEM with 2% FBS) with ANDRO or DMSO vehicle control was added and the number of cells that had migrated into the wound area after 24 h was estimated under an Olympus BX-51 microscope (Olympus, Tokyo, Japan). Cells were counted in 50 selected wound areas taken from 4 micrographs. The results were expressed as the fold change in number of cells that migrated to the wound area relative to the control culture. Every experiment was repeated four times independently.

### 5.13. Immunoblotting Assay

Protein lysates were extracted from cells by RIPA buffer and subjected to SDS PAGE and immunoblotting assay. Membranes were incubated with the primary antibodies against BMPR2 (1:1000; Merk Millipore Cat# MABD171, Billerica, MA, USA), TLR4 (1:1000; Abcam Cat# ab47093, Cambridge, UK), NF-κB p65 (1:1000; Abcam Cat# ab7970), histone (1:1000; Abcam Cat# ab13924), p-AKT (1:1000; Abcam Cat# ab81283), AKT (1:1000; Abcam Cat# ab32505), p-ERK1/2 (1:2000, Cell Signaling Technology Cat #4370, Danvers, MA, USA), ERK1/2 (1:2000, Cat # 4695, Cell Signaling Technology), p-p38 (1:1000; Abcam Cat# ab4822), p-38 (1:1000; Abcam Cat# ab7952), p-JNK1/2 (1:800; Abcam Cat# ab4821), JNK1/2 (1:800; Abcam Cat# ab85139) or β-actin (1:1000, Abcam Cat# ab8227) at 4 °C overnight followed by incubation with appropriate secondary antibodies for 1 h at room temperature. A chemiluminescent detection reagent (ECL Plus, Thermo Fisher scientific, Pittsburgh, PA, USA) was used for imaging [60].

### 5.14. Quantitative RT-PCR Analysis

Total RNA was isolated from cells using TRIzol reagent. Quantitative RT-PCR (qRT-PCR) was performed using SYBR green Universal Master Mix on an ABI StepOne real-time PCR System (Applied Biosystems, CA, USA). Previously verified primers were used including human BMPR2 forward primer 5′-CTGCGGCTGCTTCGCAGAAT-3′ and reverse primer 5′-TGGTGTTGTGTCAGGAGGTGG, IL-6 forward primer 5′-GGTACATCCTCGACGGCATCT-3′ and reverse primer 5′-GTGCCTCTTTGCTGCTTTCAC-3′, IL-8 forward primer 5′-CTGGCCGTGGCTCTCTTG-3′ and reverse primer 5′-CCTTGGCAAAACTGCACCTT-3′, TLR4 forward primer 5′-ATGGCATGGCTTACACCACC-3′ and reverse primer 5′-GAGGCCAATTTTGTCTCCACA-3′, β-actin forward primer 5′-ACTATCGGCAATGAGCG-3′ and reverse primer 5′-GAGCCAGGGCAGTAATCT-3′. Primers were synthesized by Shanghai Sangon Biotechnology Company (Shanghai, China). Comparative gene expression analysis was performed using the ΔΔCt method with normalization to the level of internal control gene β-actin. Statistical analysis was performed using a non-parametric *t*-test [60].

### 5.15. Immunofluorescence Assay

Measurements of [Ca^2+^]_i_ in live human PASMCs were performed using Fluo-3 AM (Invitrogen Corporation, Carlsbad, CA, USA) at a final concentration of 5 μM, as previously described [61]. Fluo-3 fluorescence intensity was measured (*n* = 30 to 50 cells/patients in 4 PH patients and 4 paired healthy controls).

The apoptosis of PASMCs was detected by using TUNEL (Roche, Basel, Switzerland) after serum starvation (0.1% FBS during 48 h). Nuclear counterstaining was performed using DAPI. The number of TUNEL-positive nuclei was determined by examining 5 randomly selected microscopic fields from each experimental group. The percentage of TUNEL-positive cells was calculated as follows: number of TUNEL-positive nuclei/total number of nuclei × 100% [62].

Cell proliferation was assessed by anti-PCNA and Ki67 monoclonal antibodies according to the manufacturer’s instructions. Percentages of cells nuclei positive for TUNEL, Ki67 or PCNA were determined (*n* = 30 to 50 cells/patient in 4 PH patients and 4 paired healthy controls). BMPR2 staining was performed as previously described. The positive cells (%) were measured in *n* = 30 to 50 cells/patient in 4 PH patients and 4 paired healthy controls. Alexa Fluor 488 conjugated anti-mouse IgG were used as secondary antibodies [63].

### 5.16. Data and Statistical Analysis

The data and statistical analysis comply with the recommendations of the British Journal of Pharmacology on experimental design and analysis in pharmacology [64]. All experiments were randomized and blinded. All data were normally distributed. Statistical analyses were carried out with GraphPad prism 8.00 software (GraphPad Software Inc., San Diego, USA; RRID:SCR_002798). One-way analysis of variance (ANOVA) followed by the Bonferroni post hoc test was used for multiple comparisons. Significances are represented as (* or ^#^) *p* < 0.01. In a cultured cell-based experiment, n indicates the number of experiments, while in vivo, it indicates either the number of cells measured in n animals or the number of animals only.

## Data Availability

Not applicable.

## Supplementary Materials

The following are available online at https://www.mdpi.com/article/10.3390/biom11121801/s1, Table S1: Characteristics of PH patients and control unused donors in cell culture experiments, Table S2: Comparison of hemodynamic data and right ventricle hypertrophy index in SuHx mice.

## Abbreviations

ANDRO	andrographolide
[Ca^2+^]_i_	intracellular free Ca^2+^ concentration
PH	pulmonary hypertension
PA	pulmonary artery
hPASMCs	human pulmonary artery smooth muscle cells
RV/(LV+S)	ratio of the weight of right ventricle to the weight of left ventricle and septum (also referred to as Fulton index)
RVSP	right ventricular systolic pressure
BMPR2	bone morphogenetic protein receptor type-2
IL-6	interleukin 6
IL-8	interleukin 8
qRT-PCR	quantitative real-time PCR
EdU	5-ethynyl-2′-deoxyuridine
H&E	hematoxylin-eosin staining
MTT	3-(4,5-dimethylthiazol-2-yl)-2,5-diphenyltetrazolium bromide
DMSO	dimethylsulfoxide
siRNA	small interfering RNA
Fluo-3AM	fluo-3-pentaacetoxymethylester
ROS	reactive oxygen species
NOX	NADPH oxidase
Nrf2	nuclear factor (erythroid-derived 2)-like 2
HO-1	heme oxygenase-1
