# Supplementary material for: Andrographolide Attenuates Established Pulmonary Hypertension via Rescue of Vascular Remodeling
Source: Biomolecules. 2021 Nov 30;11(12):1801. doi: 10.3390/biom11121801 (PMC8699233; doi:10.3390/biom11121801)
Supplement: Supplementary file 1 [file biomolecules-11-01801-s001.zip › biomolecules-1417495-supplementary.pdf]

Table S1. Characteristics of PH patients and control unused donors in cell culture experiments.

Patient ID	Age, y	Gender	Race	Diagnosis/Causeof Death	mPAP (mmHg)
PH-01	70	F	Yellow	pulmonary fibrosis-associated PH	42
PH-02	65	M	Yellow	pulmonary fibrosis-associated PH	41
PH-03	64	M	Yellow	pulmonary fibrosis-associated PH	44
PH-04	57	M	Yellow	pulmonary fibrosis-associated PH	36
PH-05	49	F	Yellow	pulmonary fibrosis-associated PH	45
PH-06	55	M	Yellow	pulmonary fibrosis-associated PH	40
PH-07	68	M	Yellow	IPF-associated PH	45
PH-08	60	M	Yellow	IPF-associated PH	50
PH-09	54	M	Yellow	pulmonary fibrosis-associated PH	48
PH-10	58	M	Yellow	IPF-associated PH	55
Control-01	47	F	Yellow	intracranialhemorrhage	N/A
Control-02	50	F	Yellow	anoxia of brain	N/A
Control-03	51	F	Yellow	anoxia of brain	N/A
Control-04	48	M	Yellow	cerebrovascular/stroke	N/A
Control-05	52	M	Yellow	anoxia of brain	N/A
Control-06	58	M	Yellow	cerebrovascular/stroke	N/A
Control-07	60	M	Yellow	anoxia of brain	N/A
Control-08	54	M	Yellow	anoxia of brain	N/A
Control-09	45	M	Yellow	acute myocardial infarction	N/A
Control-10	61	M	Yellow	subarachnoidhemorrhage	N/A

Definition of abbreviations: ID, identification; PH, pulmonary hypertension; mPAP, mean pulmonary artery pressure; M, male; F, female; N/A, data not available. Hemodynamic data were obtained from catheterization study performed closest to transplantation.

Table S2. Comparison of hemodynamic data and right ventricle hypertrophy index in SuHx mice.

	Control	SuHx	SuHx + DMSO	SuHx + ANDRO	*p*
pH	7.35 ± 0.065	7.37 ± 0.055	7.34 ± 0.046	7.38 ± 0.075	n.s.
PaO_2 (mmHg)	95.85 ± 7.45	55.75 ± 5.25	54.65 ± 4.85	86.55 ± 6.72	< 0.05
$PaCO_2$ (mmHg)	45.55 ± 5.15	47.65 ± 6.37	52.80 ± 7.94	48.35 ± 6.45	n.s.
HR (beats/min)	456 ± 28	440 ± 25	435 ± 20	447 ± 14	n.s.
mSAP (mmHg)	94 ± 8	96 ± 10	97 ± 4	95 ± 13	n.s.
CO (ml/min)	155 ± 12	120 ± 10	114 ± 6	142 ± 15	< 0.05
mRVSP (mmHg)	21.25 ± 2.5	42.5 ± 3.75	45.55 ± 5.65	26.75 ± 2.45	< 0.05
RV/(LV+S)	17.62 ± 1.65	30.45 ± 2.76	29.38 ± 1.5	20.22 ± 1.89	< 0.05

n = 8, * $p < 0.01$. All values are expressed as mean ± SEM. PaO_2, arterial pressure of O_2; $PaCO_2$, arterial pressure of CO_2; HR, heart rate; mSAP, mean systemic arterial pressure; CO, cardiac output; mRVSP, mean right ventricular systolic pressure; RV/(LV+S), right ventricular hypertrophy index; n.s., not significant.
